# Algorithmic classifications in credit marketing: How marketing shapes inequalities

**DOI:** 10.1177/14705931231160828

**Published:** 2023-02-28

**Authors:** Léna Pellandini-Simányi

**Affiliations:** Institute of Marketing and Communication Management, 27216 Università Della Svizzera Italiana, Lugano, Switzerland

**Keywords:** algorithms, class, classification situation, consumer data, credit, segmentation, subjectivity, inequality

## Abstract

While critical marketing studies have discussed algorithm-driven marketing’s role in governmentality, subjectivity formation and capitalist accumulation, its role in shaping class inequalities is less studied. Drawing on the performativity of marketing, ‘classification situations’ and critical algorithm studies, this paper uses the case of credit marketing to propose a twofold framework to analyse how algorithmic marketing shapes the cultural and economic inequalities of class. First, algorithms used for categorizing consumers and matching them with marketing messages and products provide access (1) to different symbolic resources and (2) to credit products with different financial consequences to different consumers depending on their categorization, which contribute to the creation of cultural and economic inequalities, respectively. Second, algorithms of financial advice devices overtake parts of consumer choice. Insofar as different financial preferences and rationalities are scripted into the devices for different client groups, these technologies constitute an additional process that affects social divisions.

## Introduction

Contemporary marketing relies on the use of large-scale consumer databases – including data from internal Customer Relationship Management (CRM) systems, online behaviour and other external databases – and the use of algorithms^1^ for key marketing tasks such as segmentation, targeting, personalized advertising and sales. Critical marketing studies analysed the new forms of governance and capitalist accumulation patterns facilitated by these systems and discussed how they simultaneously foster reflexivity and autonomy as well as the fragmentation of consumer subjects by capturing them as ‘dividual’ data points (e.g. Beckett and Nayak, 2008; Zwick and Denegri Knott, 2009; Cluley and Brown, 2015). While these studies explain how marketing discourses and practices contribute to the governance and subjectivity formation of consumers, they paid less attention to their potential consequences for the formation of social class.

These consequences are particularly pronounced in the case of financial products, such as consumer credit. Having access to a low-interest rate loan as opposed to a predatory, high-interest rate one directly influences one’s future disposable income, and as such, economic class position. While access to different credit products is largely determined by credit scoring algorithms, marketing algorithms that control which products are offered to which consumers – and which products are developed in the first place for specific segments – also play an important role. For example, if an advert-targeting algorithm targets the poor with predatory loan adverts while targeting the wealthy segments with low-interest rate offers, it contributes to making the poor poorer and the rich richer; in other words, to the deepening of economic inequalities. Segmentation and matching processes do not only control access to financial products (and to the financial benefits carried by them) but also match different consumer groups with different marketing discourses – including different subject positions called forth by these discourses – impacting not only the economic but also the cultural differences across social groups.

This theoretical paper considers this less analysed aspect of the potential effect of algorithmic marketing on class formation through the example of credit products. Applying insights from the critical marketing literature on the performativity of segmentation (e.g., Araujo, 2007; Jacobi et al., 2015), the sociology literature on ‘classification situations’ (Fourcade and Healy, 2013, 2017) and critical algorithm studies (O’Neil, 2016; Airoldi, 2022), it develops a twofold framework to analyse how specific algorithmic technologies of credit marketing may deepen, flatten and reshape the cultural and economic inequality patterns of class. The paper uses a definition of social class as relatively stable groups in society, defined, on the one hand, by socio-economic variables, such as income, occupation and education; and by the cultural variables captured through the concepts of class-specific tastes, habitus, subjectivities and values, on the other (Bourdieu, 1984; Henry and Caldwell, 2008).

The paper proposes two types of processes through which marketing algorithms potentially shape the economic and cultural elements of class. In the first type of processes, characteristic of segmentation and targeting, the consumer is the *object* of algorithmic classification and matching – a passive entity to be classified into different segments and target groups for specific credit products. These have the potential to affect the economic element of inequalities through the financial implications of different credit products for their users; as well as the cultural element of inequalities by matching different discourses with different target groups. Second, consumer choice is increasingly mediated and aided by algorithmic technologies of decision-making, especially in the case of digital financial products. In these second types of algorithmic processes, consumers are the *subjects* of algorithms: technology enables, constraints and shapes consumers’ capabilities to make financial decisions, in a Callon (1998) sense of a prosthesis and agencement. Insofar as different financial preferences and rationalities are assumed and scripted in these devices for different client groups, these technologies constitute an additional process that affects social divisions. The two processes are related (classifications determine the kind of subjectivity algorithms enact for specific consumers, and the actions taken by algorithms feed back into the classification); however, they are analytically distinct.

The paper contributes to three bodies of literature. First, it extends critical marketing research on database marketing and algorithms by complementing their focus on subjectivity formation and governmentality with the focus on how marketing algorithms may shape the cultural and economic inequalities of class. Second, it contributes to prior studies on the society-shaping role of marketing, which looked at the performative effect of marketing on social groups but paid less attention to algorithmic segmentation and to how marketing may shape the economic elements of class. Finally, the paper adds to the literature on marketing’s role in shaping contemporary debt-ridden consumer society by highlighting its effect on class and inequalities which is largely missing from existing studies. Going beyond credit markets, the broader theoretical aim of the twofold framework is to provide an analytical roadmap to think through the different ways in which algorithmic marketing technologies may shape the different elements of class inequalities across markets.

The first part of the paper reviews these three strands of literature and identifies a lack of focused theorizing of algorithmic marketing’s effect on social class. The second part discusses ‘classification situations’ (Fourcade and Healy, 2013, 2017), which is the theoretical tool used in the paper to fill this gap. The third and main part uses the case of credit marketing to develop a twofold framework to analyse how marketing algorithms shape the cultural and economic elements of class. The paper concludes by situating the arguments in relation to the literature and by outlining an agenda for further research.

## Marketing, algorithms and inequalities

### Governing consumer subjects

Recent years saw a rise in critical marketing studies on database and online marketing and the use of algorithms (e.g. Beckett and Nayak, 2008; Zwick and Denegri Knott, 2009; Cluley and Brown, 2015). Much of this research have used Foucault’s (2008) concept of neoliberal governmentality, which refers to a governance mechanism that, in contrast to prohibitive rules and laws and relying on external policing, operates ‘through the freedom and aspirations of subjects rather than in spite of them’ (Rose, 1996, p. 115). Governmentality relies on normative discourses of the ideal, ‘free’ and ‘autonomous’ subject, defined in ways that are consistent with the aims of neoliberal regimes. It encourages people to internalize these ideals and assume individual responsibility to achieve them, exercising self-governance; a process referred to as ‘responsibilization’ (e.g. Giesler and Veresiu, 2014; Yngfalk, 2016).

Combining insights from Foucault and Deleuze, this literature focused on the dynamics of subjectivity formation and governmentality facilitated by database marketing systems. Beckett (2012), for example, suggested that CRM systems and ‘collaborative marketing’ act as tools of governmentality. Calling forth the ideal subject of the reflexive, ‘sovereign consumer’, they encourage consumers to exert their autonomy through actively collaborating with producers and to self-govern through monitoring and proactively expanding their consumption choices (see also Beckett and Nayak, 2008). As Zwick and Bradshaw (2016) suggested, in these new systems, consumer sovereignty and autonomy are no longer to be controlled and disciplined but to be harnessed for economic profit. Drawing on Foucault’s notion of biopower, they proposed the term ‘biopolitical marketing’ to describe marketing activities that aim ‘to mobilize and extract value from the production of consumer communication, lifestyles, and subjectivities’ (2016, p. 3).

These studies have given important insights into how marketing systems using databases and algorithms are involved in the creation of subjectivities and governance mechanisms and into shifting dynamics of capitalist value-creation. Albeit they contain implicit takes (discussed in the next section) on how these processes may shape the cultural and economic inequalities of class, class formation has not been the focus of their analysis.

This oversight is also characteristic of the literature looking at credit marketing, the main empirical context of the present paper. Research in history has documented how marketing helped to normalize credit by providing legitimizing discourses through advertising and by developing new products that made credit accessible to the wider society (Mandell, 1990; Olney, 1991; Tucker, 1991; Hyman, 2011; Husz 2021). Studies informed by performativity and Science and Technology Studies showed the practical strategies through which credit sales agents and devices translated consumers’ everyday concerns into a need for credit and gathered information about consumers to develop credit solutions that incorporated these needs (McFall, 2014; Ossandón, 2014; Vargha, 2011). Research on the ‘financialization of everyday life’, in turn, traced how marketing, among others, played a role in the formation of responsible, self-disciplining financial consumer subjects of credit and credit scores (Langely, 2014; Pellandini-Simányi, 2021). These studies suggested that the shrinking welfare state of neoliberal regimes forced people to foot the bill of previously state-provided services from private savings and debt (Crouch, 2009). The process required a specific kind of financial subjectivity, characterized by responsible financial self-management, long-term planning, risk tolerance and openness to debt. This subjectivity has been encouraged by celebratory discourses positing financial self-management as a route to autonomy and liberation (Langley, 2007, 2008, 2009; Di Feliciantonio, 2016; Fridman, 2017). Marketing – alongside the media, the government, financial institutions and other actors – has been shown to contribute to the creation of these subjects, by providing the narratives and imaginaries as well as the actual products that presupposed and encouraged these subjectivities (Martin, 2002; Greenfield and Williams, 2007; Mulcahy, 2017).

Due to their focus on governmentality and subjectivity formation, the role of marketing in class formation has been a peripheral concern in this literature too. The question of how marketing shapes class has been addressed in turn by a different set of critical marketing studies, to which we now turn.

### How marketing shapes class patterns in the era of algorithms

The standard view of the relationship between marketing and social class patterns held that marketing merely describes and acts on pre-existing social classes but does not shape them. For example, segmentation was customarily seen as a process of discovering and describing social groups that exist independently of marketing (Kotler, 2003). Research in history, market studies and sociology have long questioned this view, arguing that marketing does not only describe but also shapes the patterns of social classes. Historical research on the origins of consumer society showed how images of domesticity, respectability and a middle-class lifestyle conveyed by marketing played a key role in the formation of the middle class (McKendrick and Brewer, 1982; Haynes, 2010). Marketing has also been credited with contributing to the differentiation of classes into value- and consumption-based lifestyle groups and subcultures by providing the cultural imaginaries around which these groups are organized (Giddens, 1991; Shields, 1992).

Zooming into marketing practices, interdisciplinary market studies suggested that segmentation is a performative process, through which new social groups may be created and existing ones reshaped (Araujo, 2007; Jacobi et al., 2015; see also Sunderland and Denny, 2011 for a critique). For example, Ariztía (2014) traced how marketing agencies described the C3 segment and then actively contributed to its emergence through the creation of discourses, lifestyle imaginaries and goods. As Cluley (2019) aptly summarizes: segmentation ‘orders consumer markets into manageable chunks that can become real as brands design new products, communicate new meanings and promote offers to particular segments’ (p. 48).

Most of these studies, however, were written before algorithms became central to marketing and paid scarce attention to them. The increasing use of consumer datab – either from internal CRM systems or from external sources, such as online data – allowed for new ways of segmenting consumers to match them with products and services with the use of algorithms (Zwick and Denegri Knott, 2009; Cluley and Brown, 2015). In credit marketing, for example, segmentation and targeting are often done through algorithmic scoring that categorizes people based on their scores and decides who to target with an online advert or with an offer in a bank branch based on these categories. Algorithms also drive financial advice devices, such as chatbots, and decide what advice should be given to a customer interested, for instance, in online mortgages.

How does the use of algorithms affect the way marketing performs class? While this question has not been analysed by critical marketing scholars, critical algorithm studies provide useful transferable theoretical insights. These studies analysed how predictive and scoring algorithms – used, for instance, in school entry exams, allocating welfare benefits, or crime prevention – categorize people into different groups and allocate different scores to them, which, in turn, determine their access to university, welfare benefits or the length of their jail sentence when committing a crime (Amoore and Piotukh, 2015; O’Neil, 2016; Airoldi, 2022). They suggested that algorithmic classifications, depending on which variables they use, how they form the categories, and how they match predictions with the categories, may deepen, flatten or reproduce social inequalities (Burrell and Fourcade, 2021; Airoldi, 2022). While marketing algorithms are often mentioned as examples, very few studies analysed their role in the production of inequality. One exception is sociologist Massimo Airoldi (2021), who showed that music and movie recommendation algorithms reproduce taste structures, and as such cultural inequalities. He suggested to analyse machine learning algorithms as acting on and acting out a particular habitus (Airoldi, 2022).

The critical marketing studies on databases discussed in the previous section, albeit focusing on subjectivity formation, governance and capitalist accumulation, also provide – often implicit – takes on how algorithmic marketing sorting and matching practices may shape the patterns of social groups. We can identify two main, contrasting takes in this literature. The first suggests that CRM systems and databases, equipped with more fine-grained knowledge of consumers, perform social groups even more powerfully than in the era of traditional segmentation (Pridmore and Lyon, 2011). For example, Beckett and Nayak (2008) and Beckett (2012) describe how the Tesco CRM system fosters normative identities and governs consumers by encouraging them to associate themselves with these identities. This involves a reflexive engagement with their consumption patterns, shaped by normative recommendations from TESCO on what similar consumers buy – tying consumers to the norms of the groups of ‘similar consumers’ identified by the CRM system. This analysis implies that digital data allows for even more powerful group-formation processes. As Hietanen et al. (2022) suggest: ‘the practice of segmentation itself is always an aggressive move to perpetuate symbolic class differences. Now it would seem that class difference will be fully normalized – automatically preconfigured and perpetuated as a continuous modulation of the potential to be included and excluded’ (p. 9).

The second, contrasting view suggests that whereas traditional marketing performed social groups by encouraging people to adopt a self-identity tied to specific groups (such as subcultures, class or lifestyle), digital segmentation and targeting works with the opposite logic. As Cluley and Brown (2015) explain, using Deleuze’s concept of the ‘dividual’, online marketing innovations ‘treat consumers not as fixed individuals but as dividualised consumers – that is to say, collections of data that can be exposed, dissected and segmented into new marketable groups’. In a similar vein, Cheney-Lippold (2011) developed the concept of ‘algorithmic identity’, which refers to the identity inferred by web-analytics companies based on our web-surfing habits. This identity consists of data points that reliably predict our purchasing behaviour, grouping us with others with similar data points, and is constantly reconfigured in the light of feedback. Zwick and Denegri Knott (2009) suggest that in this type of segmentation, the logic is no longer to match products to given consumer groups, but the other way round, to create consumer bundles from the database that match an existing commodity: ‘rather than adjusting the functionality of commodities to match consumer desires, marketers can now modulate, at very little cost and in real-time, the functionality of consumers to match an existing commodity’ (p. 236).

The segments created this way no longer aim to capture consumer personalities, only some of their dividual characteristics. Further, the segments are rapidly shifting, partly because the target groups are re-made depending on the client companies’ different marketing objectives, and partly because the consumer data is constantly updated based on the consumer’s responses to previous marketing messages. If marketing no longer uses social groups for segmentation, only ephemeral groupings along different variables in the dataset, it implies that it no longer performs social groups in real life either.

While these works have provided important insights into how the processes through which marketing shapes social groups may change with the use of databases, their theoretical focus on the production of subjectivity and new forms of governance precluded the theorization of how marketing algorithms shape class. Moreover, due to their empirical focus on FMCG (Beckett, 2012) and cultural products (Airoldi, 2021), they analysed almost exclusively the cultural element of categorizations: the way databases classify and foster similar ways of thinking and acting among people categorized as similar. Marketing’s effect on the economic variables of class is less explored. To theorize these processes, this article looks at the marketing of credit products and draws on the economic sociology literature on how credit eligibility algorithms shape the economic elements of class, discussed in the next section.

### ‘Classification situations’: How credit scoring shapes inequalities

Access to consumer goods has always affected people’s ability to belong to a specific social group. Goods serve as tokens and expressions of class tastes and identities (Bourdieu, 1984), which means, for example, that someone who does not have access to the latest luxury fashion may be excluded from the upper class. However, access to *financial products* affects people’s access to specific class positions in a more fundamental way: by directly influencing their future disposable income and wealth. Having access to a low-interest rate loan as opposed to a high-interest rate one, objectively makes a borrower better off. Conversely, being denied a mortgage excludes the person from jumping on the ‘property ladder’ and from accumulating housing wealth.

This latter insight forms the core of Marion Fourcade and Kieran Healy’s (2013) argument on ‘classification situations’. Their starting point, drawing on Max Weber, is that social class, instead of being defined as a fixed position in the system of production – captured, by Marx’s (1977) definition of class based on capital endowments or by definitions based on income, education and occupational position (Henry and Caldwell, 2008) – should be defined as *groups facing similar life chances*. Which people have similar life chances is not self-evident and cannot be established a priori, but only empirically: by identifying the processes that create these similar life chances. If in a society, resources are distributed according to hair colour, then people of the same hair colour experience similar life chances, and thus form a class; if resources are distributed based on education level, then people of similar education form a class. The logic can be extended to situations with multiple class-forming factors. In their framework, every grouping that describes similar life chances is a ‘class’. To avoid confusion, we will distinguish ‘class’ defined this way from the traditional definition that considers class as people of similar wealth, income, education and occupation by referring to the latter as ‘traditional markers of class’.

Fourcade and Healy call the categories that determine people’s access to goods, services and opportunities, ‘classification situations’ (2013, p. 561). They argue that in market societies, market institutions play an important role in classifying people into different ‘market categories’, which define their access to the market itself and within it, to specific goods. Unlike in previous periods, in today’s market society, classification situations increasingly take the form of scoring, based on which people are classified into different categories that define their access to valued goods – and consequently, to life chances.

They distinguish two main types of classification situations. *Boundary classifications* decide who can and cannot participate in a market, whereas *within market classifications* decide with which conditions one can participate. Their prime example is credit scoring technologies. These technologies establish, first, who can get specific credit products (boundary classification). For example, redlining is – or used to be – a practice of excluding inhabitants of specific neighbourhoods from mortgages (Aalbers, 2011; Dwyer, 2018). Second, they establish with what conditions – interest rate, collateral value, guarantors, etc. – people can get credit (within market classification), depending on the group into which they have been classified, typically through credit scoring (Marron, 2007). Fourcade and Healy (2013) argue that the sorting mechanisms of credit scoring do more than simply describe differences between high-risk and low-risk borrowers. Rather, they act as vehicles for producing inequalities by granting access to people to better or worse financial opportunities. The classification affects one’s objective life chances – hence, the groups produced through the classificatory mechanism are ‘classes’ in a Weberian sense explained above.

We note here that these concepts can usefully be related to other theories that describe processes of inclusion and exclusion, such as inclusion through legitimacy (e.g. Muniz and O’Guinn, 2001). On the one hand, they add to them by analytically distinguishing processes that (1) block access and (2) that provide access, but to a different or to a limited membership. On the other hand, the focus of the concept of ‘classification situations’ is narrower in that it refers to *class*-ification situations, that is, to inclusion and exclusion that shapes social class.

As we are discussing access to *credit* here, it is worth pausing to consider whether this access is indeed a privilege that leads to better financial chances. As Arnould and Press (2019) point out, while market access to goods is generally considered a good thing – a privilege – in specific cases it can also be the opposite, for example, a means of neo-colonial exploitation. One part of the literature considers access to credit as an unequivocally good thing. Credit allows people to access consumer goods, and even to increase their financial wealth by jumping on the ‘property ladder’ with a mortgage. In this reading, access to credit is part of the ‘democratization of finance’ (Erturk et al., 2007), akin to a citizen right that allows people to participate in society and maintain dignity (Wherry et al., 2019). Another part of the literature, in contrast, considers credit a bad thing both at a personal and at a structural level (Hyman, 2011). At a personal level, credit is seen to deteriorate one’s financial chances, potentially leading to a debt trap and eviction, alongside health issues and distress in everyday life and relationships (Drentea and Lavrakas, 2000; Han, 2012; Porter, 2012; Desmond, 2016).

The point of Fourcade and Healy (2013) is that credit *per se* does not increase or decrease life chance; certain forms of credit, which grant favourable conditions increase them (in the sense that they allow the borrower to acquire goods earlier without threatening her financial well-being and to accumulate wealth), while other forms of credit, with less favourable and even predatory conditions, decrease them. This is why their focus is on classificatory mechanisms that determine who gets credit and with what conditions – and the relative, rather than absolute, financial consequences of these classificatory processes.

This stratifying effect is best illustrated by considering how different credit eligibility methods classify people and how these differences have affected inequality. Before the rise of credit scoring technology, credit eligibility was done by humans: bank officers, assessing the applicant’s morality, reliability, frugality and diligence (Muldrew, 1998). Examining these evaluations as classification situations and their consequences for inequality suggests, on the one hand, that being done by a sole human evaluator, they were subject to several biases, such as racist and classist views (Fourcade and Healy, 2013; O’Neil, 2016), and were likely to reproduce and deepen existing inequalities. Indeed, formal credit scoring was introduced in the United States as a way to diminish the biases of personal evaluation (Guseva and Rona-Tas, 2019). On the other hand, they were based on the intimate knowledge of the applicant, which allowed the credit evaluator to override class considerations and give a loan with better conditions to a person who the evaluator deemed trustworthy, even if the actual income and wealth of the applicant would not have justified it (Finn, 2003).

Modern credit assessment uses models that are developed based on other people’s past behaviour (Rona-Tas and Guseva, 2018; Guseva and Rona-Tas, 2019). Hence, it is a ‘prediction of the future behaviour of a loan applicant based on how comparable people behaved in the past’ (Rona-Tas and Guseva, 2018, p. 63). The characteristics that are used to build the prediction model are of crucial importance because they delimit on what basis people can be ‘comparable’, that is, which characteristics will be used to assess one’s credit request. They define how the model can reflect, ‘see’ society (Fourcade and Healy, 2017) and how it may deepen, flatten or reshape exiting inequalities.

For example, credit assessment based on demographic characteristics, such as gender, age, living location, income and occupation (Rona-Tas and Guseva, 2018; Guseva and Rona-Tas, 2019), deepens traditional class inequalities. This is because living location, income and occupation are key markers of traditional social classes, and if they are used in the models that establish eligibility and conditions, they are likely to result in higher interest rates for the poor and lower for the rich – under the neural language of ‘risk premium’. In contrast, a different, widely used method of scoring based on the person’s very own past behaviour – how diligently the applicant paid previous credits, bills and other financial obligations (Guseva and Rona-Tas, 2019) – rather than his or her social category, could, in theory, flatten and even reshape inequalities, as the diligently paying poor borrower should get the same conditions as a diligently paying rich one. Indeed, this system emphasizes individual agency and presents favourable versus unfavourable conditions as rewards and punishments for the moral character (Marron, 2009). However, this ignores the fact that payment behaviour, such as late payment, is largely influenced by economic conditions. This way, even behavioural markers may act as proxies of traditional markers of class. In fact, they may be even more accurate proxies than those based on income and occupation, as they tap directly into financial vulnerability central to class.

Further, behavioural markers are rarely used in themselves to define eligibility: income and wealth impose constraints for several credit products (e.g. mortgages) in the form of minimum collateral values and debt-to-income ratios, defined by regulation and banks’ prudency policies. This means that certain debt products – arguably, the most beneficial ones, such as mortgages as opposed to payday loans – tend to be out of reach for the poor, irrespective of how well they behave, reproducing rather than flattening existing class differences.

It is important to note here that these effects stem from the fact that eligibility algorithms are generally programmed to manage the risk of non-payment by matching higher risk with a higher price. The algorithm can also be programmed for an inequality-reducing effect, for example, by offering better loan conditions to the poor. Alternatively, it can be programmed in a way that generates new forms of inequalities, for example, a faulty algorithm that gives better conditions to people whose name starts with M. It is not possible to make a general statement on how credit scoring algorithms affect inequality; rather, in the case of each specific algorithm, we need to think through how the classification mechanism reflects, often unconsciously, existing socio-economic divisions and how it matches the resulting categories with products that offer higher and lower economic rewards.

### How algorithmic credit marketing shapes inequalities

Eligibility algorithms are key classificatory techniques that shape social class. However, they are not the only ones. Financial providers’ marketing departments also use various algorithmic processes of sorting consumers and matching them with financial products and marketing messages. Identifying consumer segments, establishing which consumers to target with customized advertisements, and whom to offer a payday loan or a flexible mortgage solution are all examples of sorting and matching practices used in the marketing of credit products. Matching consumers with financial products is not only done via targeted messages but also through financial advice chatbots, comparison websites and other apps that use algorithms to match consumers with the credit best suited to them.

In what follows, we use the theoretical tools of Fourcade and Healy to understand how these algorithmic marketing tools may shape social classes. We will propose two main processes, which we discuss below. The first, typical of classification processes used in marketing, similarly to eligibility algorithms, sort people into segments and match them with specific products and messages. In these technologies, the consumer is the *object *of algorithms because people are classified by algorithms. In the second, characteristic of algorithms of financial advice and choice devices, which operate by co-constructing consumers’ subjectivity, the consumer becomes the *subject* of algorithms.

## Consumers as objects of algorithms: Segmentation and targeting

### Credit scoring versus marketing scoring

The mechanisms through which marketing sorting affects social groups and inequalities are partly similar to credit scoring algorithms. They classify people and provide different degrees of access to products with higher and lower economic rewards based on the classification. Sorting mechanisms, such as segmentation, define the groups for which products will be developed in the first place and they are used to devise the properties of new products (Lopes, 2013) – including their economic benefits. For example, product development teams may decide to develop a reward-based credit card product for an upper-middle class target group; while another credit product without proof of income, yet high-interest rate for a lower-class group. Marketing decides the distribution channels, including which product to offer in bank branches, online or via third parties. It is also in charge of the communication channel and message: whether a new loan will be advertised on billboards accessible for everyone or if it will be offered via personal agents; who will receive a call with a favourable loan offer, and on whose computer screen a tempting payday loan offer will pop up (Mierzwinski and Chester, 2013; Cluley and Brown, 2015).

These marketing classifying practices are similar to credit scoring in that they influence the access of people to credit products with higher and lower economic rewards – and as such, they may deepen, flatten or rewrite inequalities. However, they also differ from credit scoring. First, credit scoring’s sole purpose is to minimize the banks’ losses by managing risk. They match poor consumers with a higher-rate loan due to the intrinsic logic of risk-based pricing. In contrast, marketing’s purpose is client satisfaction which leads to long-term relationships, market share and profit. Marketing needs to offer products that make not just the bank, but also clients happy. This means that marketing, in theory, could develop products that are more favourable for the poor if this is what is required to acquire and keep these clients (within the constraints set by the risk department). This means that marketing sorting is less pre-determined to deepen inequalities than credit scoring.

Second, marketing scores do not exclude groups the same way as credit scoring does, which explicitly denies people specific products. Yet they may still significantly hinder access of specific people to specific products. For example, economic geography scholarship on financial ecosystems shows that a key obstacle for poor people to access financial products is their lack of access to the distribution channel – to the bank branches (Leyshon and Thrift, 1995). Hence, marketing decisions of distribution channels affect who gets access to a product. This is even more the case with sales algorithms for targeted, customized product offers. Indeed, regulators and legal scholars proposed that data brokers and marketing-list businesses that provide targeting services should be treated as Credit Rating Agencies from a regulatory point of view (Mierzwinski and Chester, 2013). The reason is that what these ad-matching algorithms do is very similar to credit rating: they score consumers and use these scores to decide who gets a particular offer and who does not.

Finally, unlike credit scores that allocate merely access to products, marketing categorizations also allocate access to specific marketing discourses. Depending on how one is categorized, one is targeted with different adverts and marketing messages. Thinking further the arguments reviewed in the first part on subjectivity formation, if CRM systems and marketing tools promote specific kinds of subjectivities and self-discipline, then being exposed to different marketing discourses – depending on how one is categorized – may promote different economic rationalities marked by different preferences, motivations and tendencies. For example, inviting wealthy consumers to adopt an investorial subjectivity in their mortgage choices, while promoting a ‘Carpe diem!’ mentality for the poor in their credit choices may foster differences in financial subjectivities across classes. Further, taking seriously the Foucauldian point that subjectivity develops through practice, by offering different products, marketing offers different practical tools to engage in specific subjectivity formation projects. This means that categorizing algorithms may act as tools of differential subjectivity formation across social groups, shaping the cultural element of class.

### Marketing scoring shaping inequalities

In this section, we focus on how marketing sorting and scoring may shape economic inequality. We will first consider the sorting processes of segmentation, positioning and targeting used for the *development of new products*: the design of marketing and distribution channels, product propositions and messages. Then we will turn to the sorting processes used to determine which of the *existing products* are offered to new and existing clients, via direct messages, online ads, in the bank branches, and so on.

Studies on how banks segment their retail (non-business) clients for *developing new products* reveal three main types of segmentation, based on (1) demographics, (2) past financial behaviour and (3) needs/attitudes (Bailey et al., 2010; Fiorio et al., 2014; Krenn, 2017). Segmentation based on *demographics* typically includes wealth – dividing clients based on their net worth – income, age and geography (Bailey et al., 2010; Fiorio et al., 2014). It usually uses data from census reports, other third-party databases and internal CRM systems (Fiorio et al., 2014). Segments are used for the core marketing strategy (Bailey et al., 2010), which includes identifying the target segment for a new product and developing the key product characteristics.

This segmentation, due to its reliance on demographics that overlap with traditional markers of class – income, education, occupation and living location – clearly reproduces traditional class categories. Whether this deepens or flattens inequalities, however, depends on what kind of products are developed for the different segments. For example, McKinsey suggests that for the poor segment, banks should develop products that have low, transparent and clearly explained fees, and products that help them to manage money better by setting credit limits and offering money-management tools (Fiorio et al., 2014). If financial institutions would follow this advice, traditional class-based segmentation could, in principle, flatten social inequalities.

However, the actual products developed for the poor suggest that this is rarely the case. Many of these products prey on poor consumers’ vulnerability and low financial literacy, charging often predatory fees and encouraging, rather than preventing over-indebtedness and high-risk borrowing (Squires, 2004; Deville and Van der Velden, 2016; Cochoy et al., 2017a). In contrast, to retain ‘high net worth clients’, banks offer them custom-made, individual, advantageous products and advice (Lazarus, 2012; Krenn, 2017). This contributes further to the deepening of inequalities.

The second segmentation principle for new products is based on *past financial behaviour*, using credit bureau reports and CRM data on spending and payment behaviours (Denecker et al., 2014). This segmentation could crosscut traditional class differences if the financially ‘well-behaved’ poor would be in the same segment as the ‘well-behaved’ rich and/or if products with similar economic rewards would be offered to each segment. However, here too, the reality tends to be the opposite. This segmentation is rarely used alone, but in combination with demographic data (Bailey et al., 2010; Denecker et al., 2014) – meaning that the well-behaved poor are never in the same segment as the well-behaved rich, with less advantageous products being offered to them. For example, this segmentation forms the basis of categorizing consumers into groups of ‘transactors’, ‘revolvers’ and ‘subprime’, which are matched with product offers of ‘reward’, ‘revolving’ and ‘subprime’ credit cards, respectively (Denecker et al., 2014). Given that the ‘reward’ card offers the most benefits, while the ‘subprime’ the least with the highest interest rates, applied this way, this segmentation too, tends to reproduce and deepen existing economic class divisions.

The last segmentation principle of *need-based or attitudinal* segmentation is based partly on demographic data and partly on people’s attitudes to money, shopping, saving and spending as well as financial behaviours. It is used for developing core product characteristics, the product proposition, media messages and communication channels (Bailey et al., 2010; Fiorio et al., 2014). While this segmentation too, promises to depart from the traditional category of class, in practice, it large overlaps, disguising the structural effects of class in the agentic language of attitudes. For example, McKinsey’s (Fiorio et al., 2014) need-based segmentation that identifies five segments suggests that consumers of the wealthiest, ‘Prosperous and content’ segment ‘love rewards’; hence, issuers should position credit cards for this segment

not merely as a spending instrument but as a tool that facilitates financial success through ease of use. For instance, if a credit card could offer a means of steering a large purchase straight into a low-rate instalment loan, it could meet this segment’s occasional borrowing needs without the stigma or higher interest rates associated with revolving credit. (Fiorio et al., 2014, p. 13)

Offering rewards is explained as the right value proposition for the needs of this segment, akin to the beneficial low-interest rates, which are justified by the segments’ aversion to the ‘stigma’ of high-rate revolving credit. This suggests that taking a high-interest rate, disadvantageous credit, after all, is just a matter of preference, rather than financial deprivation. Need-based segmentation is class-blind: through the language of needs and customer satisfaction, it offers advantageous products to the rich and less advantageous ones to the poor without using the language of class. This class-blindness, however, does not prevent it from reproducing and deepening traditional class inequalities; only from seeing it.

While the sorting methods discussed so far are used for the development of *new products* and the marketing strategy, other sorting algorithms are deployed as part of ‘executional segmentation’ (Bailey et al., 2010) to decide who to target with personalized offers of the *existing product range*. These offers may be directed to existing clients for cross- and up-selling, for example, via direct mail, phone (through telemarketing or during incoming calls to the call centre) or personal advice in bank branches (Vargha, 2011); but also to non-clients via offline and online adverts and offers (Mierzwinski and Chester, 2013). These algorithms match specific clients with specific offers from the existing product portfolio; hence, they also act as gatekeepers and facilitators of access – with consequences for class differences and inequality.

The essence of these sorting mechanisms is ‘predictive marketing’ (Kotras, 2020), which seeks to identify the characteristics of potential clients that are likely to purchase a product and the characteristics of those who should be avoided as clients – such as people who are likely to commit fraud (Kotras, 2020). To identify these characteristics, financial service providers or third-party data analysts first build a statistical propensity model based on a database of the past behaviour of existing clients. For example, Barclays Bank’s propensity modelling, which is used for the algorithm that decides which products sales representatives should offer to a specific client, is built on a database that contains clients’ contact and transactional history from current accounts and credit cards from Barclays’ own CRM system and data from external data providers on ‘customers’ lifestyles, finances, careers, spending habits and travel’ (Bailey et al., 2010, p. 230).

The propensity model based on the database is typically a regression model with product use, fraud, churn – or whatever the bank wants to predict – as a dependent, target variable, built with the objective of identifying key independent variables that predict the target variable. For example, the purchase of a payday loan may be predicted with high confidence based on a set of demographic variables, such as income and prior behaviour, such as late payment, or online behaviour, such as a prior Google search for ‘quick loan’. The independent variables that predict well the dependent variable in the database are bundled together into a propensity score (a high score indicating that the person is likely to perform the predicted behaviour).

In the next step, an algorithm is built that calculates potential clients’ propensity scores and offers people specific products depending on their scores. To calculate the score, a monitoring and scoring system for potential clients needs to be in place. For example, CRM systems monitor clients’ late payments and expiring mortgages (Bailey et al., 2010), while online recommendation algorithms monitor people via the cookies that record their web browsing history^2^ (Cheney-Lippold, 2011; Mierzwinski and Chester, 2013; Cluley and Brown, 2015). When a monitored client’s score matches the algorithm’s criteria, the given, customized product offer is made for the client. For example, a client with a late payment will receive a call with a credit offer or someone who is browsing property websites will get a mortgage offer on her computer screen (Bailey et al., 2010; Mierzwinski and Chester, 2013). These models are updated in the light of new data – including the success of the offers (Vargha, 2017) – which is part of the concept of ‘machine learning’ (Kotras, 2020).

The kind of past data on which these propensity models are built, the new data they use to score potential clients, and the algorithms that match them with specific products based on their score are central to whether and how they reproduce and deepen inequalities. For example, an anonymous bank described by Kotras (2020) uses propensity models to predict ‘financial difficulties’ such as late payments and overdrafts to offer clients specific credit products. To develop this model, the data scientists used internal and external data, such as economic data and zip codes. Based on the dataset, they identified a set of variables to predict who is likely to have financial difficulties: ‘a set of relatively classic variables, including each client’s turnover (and its possible decline), as well as the activity of its economic sector and geographical area’ (2020, p. 6). In this case, the data used to develop the model was heavily focused on classic class variables (economic data and location). Financial difficulties, not surprisingly, were found to be predicted by these ‘classic’ class indicators.

Similarly, Barclays Bank’s models identify specific ‘triggers’ on its clients’ accounts that indicate a potential credit need, such as late payments or a large credit card withdrawal; as well as ‘events’, such as a mortgage coming to an end, getting married or having children as predictors for upcoming mortgage needs. These events and triggers indicate, for example, that the client may respond to a credit card or a mortgage offer, hence that bank monitors them in its internal CRM system (Bailey et al., 2010). To arrive at the right product offer, however, it combines the data with clients’ creditworthiness to decide, for example, ‘whether a loan would be an appropriate offer for a customer who has just been charged a late payment fee or an overdraft extension’ (2010, p. 235).

What these two examples suggest is that albeit propensity models for credit products may not look for traditional markers of class, they often end up with categories that closely reflect them. This is because, first, the data that they use are directly (income, wealth, geographic data) or indirectly (e.g. payment difficulties) related to class. Second, to arrive at the actual offer, creditworthiness needs to be taken into consideration, which includes class variables. This means that the same ‘trigger’ often results in a different product offered to the rich versus the poor – for instance, a lower interest rate loan to the creditworthy rich and a higher interest rate overdraft to the poor.

The matching process for targeting online offers to new clients also uses a propensity model developed based on the same principles: third-party companies acquire data on existing financial consumers, including offline data, such as credit records, and online data on web-surfing behaviour (Zwick and Denegri Knott, 2009; Cheney-Lippold, 2011). However, unlike existing clients in the CRM system, web surfers are anonymous, hence the model needs to be applied to potential new clients based on data solely on their online behaviour: prior searches, clicks on adverts, social media use and so on, with the help of cookies that track the user across different websites (Cluley and Brown, 2015), which are less likely to include direct class indicators such as income.

The resulting matching algorithm may be as simple as one that says that people who click on the ‘Get credit quickly!’ ad are more likely to be interested in payday loans, so people with a cookie showing that they clicked on the ad should be targeted with a payday loan. But they may be also complex, describing propensity segments along several variables, including interests inferred from their web history, the data they shared with Facebook (which includes demographic data, such as age), geolocation data or screen resolution, which can be used as a proxy for income (Deville and Van der Velden, 2016).

Depending on the data used for modelling and profiling new clients, this method can also have different implications for inequality. If the propensity model and the collected data focus on traditional class variables and their proxies and matches the poor with disadvantageous credit offers, it is likely to deepen inequalities. However, this is not an inevitable consequence of using online behavioural data. Models using online behaviour have, in principle, more potential for cross-cutting inequalities because they do not have access to direct class data, including income and wealth, only to indirect one, inferred from online behaviour. If a poor and a wealthy consumer behaves similarly online, these models are unable to distinguish between them (unless consumers give out their financial data, for instance, when using a loan comparison app).

### Consumers as subjects of algorithms: The digitalization of choice

In financial eligibility assessment and market segmentation, the consumer is the *object* of algorithms: a passive participant to whom matching ‘happens’, being sorted into categories. In the previous part, we showed that how one is categorized influences firstly, one’s access to financial products with different financial characteristics, and secondly, one’s exposure to specific marketing discourses and practices. We argued that marketing categorizations are thus ‘classification situations’ that can potentially shape both the economic and cultural elements of class.

Whereas in these cases algorithms are used to categorize consumers, algorithms in credit marketing are also used as consumer decision-aiding devices. While discourses contribute to different subjectivities and actions in a subtle way – offering, rather than enforcing them on consumers – decision-aiding algorithms play a more direct role in mediating consumer action. Comparison websites and apps with integrated choice-aiding devices, such as financial advice chatbots, do not simply call forth specific subjectivities and actions but take over part of the decision-making. They calculate for consumers, pre-select choices for them and advise them on which products best suit their needs. In these cases, consumers are not only classified but specific consumer actions and thought processes – such as calculations or defining one’s financial aims and needs – are done by the algorithm. In these cases, the consumers are the *subject* of algorithms. What we mean by this is not that consumer subjectivity as a whole is mediated by algorithms, but that certain aspects of subjectivity are; in this case, economic subjectivity, that sociology captures through notions of the economic habitus while economics through concepts such as risk tolerance, debt tolerance, financial time horizons and (bounded) rational calculation.

To theorize this process, we draw on Callon’s (1998) influential introduction to *The Laws of the Markets* which argued that for rational, calculative action to take place, people do not need to be rational and calculative. Rather, they need ‘prostheses’, that is, (material) devices that enable, or even accomplish calculation and rational choices for them. Think of a mortgage comparison website: after answering a few basic questions, the website’s algorithm calculates the best choice for you. You are able to make a rational, calculative choice without being rational or able to perform the calculations yourself. The key argument of Callon’s approach is that material devices, instruments, dispositives prefigure and perform particular forms of rational subjectivity and action (e.g. Callon et al., 2007; McFall, 2014; Cochoy et al., 2017b; Cochoy et al., 2017a). They do not simply encourage actions of a pre-existing subject; rather, agency itself is a result of human-non-human assemblage. Particular devices allow for different forms of agency; they function as ‘agencement’. Devices are thus seen as key in the production of rationalities: they reflect specific assumptions of the subject; which they do not merely describe, but performatively produce through their script. Partly based on these insights, recent marketing scholarship has reflected on the changes in decision-making aided by digital technology, especially in online environments. These works suggest that digital devices increasingly make decisions for consumers – or at least, they co-construct them, captured by notions of ‘exogenous cognition’ (Smith et al., 2021) and ‘technology-shaped choice processes’ (Dholakia et al., 2021).

We can apply this point to the way consumers choose financial products, which are often aided by targeted, algorithm-generated advice. Studying mortgage selling in bank branches, Vargha (2011) argued that rather than simply offering particular products to clients’ pre-existing financial needs, the very process of financial demonstration creates the client as an economic agent with specific preferences and economic calculations: ‘(a)s sellers perform products interactively with clients, consumers’ needs appear. Financial demonstrations yield different consumers-with-preferences’ (2011, p. 215).

This insight is even more relevant in the context of digital financial advice devices, which take over more and more decisions from the consumer subject and delegate them to the algorithmic technologies used by these apps and websites. The technical features (the user interface, sliders, choice options as well as the algorithms making recommendations) of specific financial apps and websites invite and prefigure particular choices, rationalities and interactions. For example, studies on the ‘fringe’ finance of payday loans documented how digital credit interfaces foster indebtedness by setting higher default borrowing amounts and providing a smoother borrowing experience (Ash et al., 2018; Langley et al., 2019).

At a more general level, decision-aiding algorithms need to make assumptions about the goals, risk preferences, time horizons and financial goals of their users. For example, if the algorithm assumes that the goal is to get the highest loan amount possible (which implies higher repayment amounts and, often, higher interest rate and higher risk), it will give different advice than if it assumes that the goal is to maximize long-term profit (which implies that repayments need to be compared to the revenues generated through the investment financed by the loan). Ideally, the algorithm should not assume but ask for these preferences. But a key point of choice-aiding algorithms is to provide a smooth, even gamified experience. They are not supposed to ask difficult questions, or a long list of questions. Even, they are tasked with assessing user preferences that users themselves did not even know they had – such as their risk tolerance or planning horizons. This means that in practice, choice-aiding algorithms do make assumptions. For example, the algorithm used in bank branches to aid mortgage choices is often programmed with the assumption that clients want to get the highest mortgage amount possible, even if it means that they need to pay back more, for longer and bear more risks. Clients that prefer to have a smaller mortgage in order to minimize repayments and risks have a hard time asserting their preferences against these default assumptions (Pellandini-Simányi and Vargha, 2019). Algorithms, left to their own devices, act out the financial rationalities that are assumed by their decision rules.

Combined with algorithms that categorize and match consumers, decision-making algorithms may prefigure different choice processes and financial rationalities for different client groups. For example, Deville and Van der Velden (2016) showed that credit providers use geographic and screen resolution data (used as a proxy of the quality of the computer and thus of the user’s wealth), among others, to customize the credit application interface, including the default amount and the best offers. Payday loan apps typically assume that their users are in dire financial need who only care about maximizing the loan amount, while mortgage loan apps used by wealthier segments increasingly include functions for calculating the buy-to-let profit that can be achieved by borrowing. In these cases, not only specific conditions but specific rationalities are matched with categories of consumers. This process may further contribute to traditional class divisions, by encouraging and even performing different financial rationalities and behaviours for different categories (which, in turn, may influence objective life chances).

## Conclusion

While critical marketing studies discussed database and algorithm-driven marketing’s role in governmentality, subjectivity formation and capitalist accumulation (e.g. Beckett and Nayak, 2008; Zwick and Denegri Knott, 2009; Cluley and Brown, 2015), its role in shaping class inequalities has been less studied. Drawing on the critical marketing literature on the performativity of segmentation (e.g. Araujo, 2007; Jacobi et al., 2015), critical algorithm studies (O’Neil, 2016; Airoldi, 2022) and economic sociology arguments on ‘classification situations’ (Fourcade and Healy, 2013, 2017), this paper drew attention to this less studied angle. Using the case of algorithms in credit marketing, it proposed a twofold framework to analyse how algorithmic marketing tools may shape the cultural and economic elements of social class.

The framework outlined two main types of processes through which marketing algorithms shape class depending on whether they are used for categorizing and matching consumers with products or for aiding consumer decision-making. In the first one, the consumer is a passive *object* of algorithmic classifications. These categorization algorithms that sort consumers for credit products through segmentation and targeting have the potential to shape, firstly, economic inequalities. This is because different financial products with different conditions have concrete, economic costs and benefits for their customers. Marketing categorizations that provide different access to financial products with higher or lower economic costs and benefits thus potentially shape the objective economic life chances of their users, and as such, economic inequalities. For example, by developing and targeting a payday loan to a poor consumer segment and a favourable mortgage to a wealthy one, marketing may contribute to the deepening of existing economic inequalities. This imbues the marketing of financial products with a potential for a more direct social stratificatory power than it has in other markets.

Secondly, marketing categorizations also influence the allocation of marketing messages across different groups. This means that depending on how one is categorized by marketing algorithms, one is exposed to different discourses, which may call forth different subjectivities. As a result of the categorization, scoring and matching processes, *qualitatively* different consumers with different forms of rationalities, economic subjectivities and behaviours are performed. Through this mechanism, algorithmic marketing classifications shape the cultural element of class by defining patterns of access to symbolic resources.

Algorithms are not only involved in categorizing and matching but they increasingly penetrate the consumer choice process itself through digital advice and nudging, acting on the consumer as the *subject* of algorithms. Choice-aiding devices are scripted with specific financial rationalities and steer users towards particular behaviours and subjectivities. Given that these devices are also targeted at specific categories and structure advice differently for different user categories, they represent the second, additional process through which marketing’s stratifying effects play out: by fostering different, classed economic rationalities and behaviours.

The two types of processes are analytically distinct, yet related. Categorization algorithms may determine what kind of subjectivity choice-making algorithms perform for specific consumers. In turn, the choices made with the help of choice-making algorithms show up as ‘prior purchase behaviour’ in the system, constituting the basis for future categorizations and a potential self-reinforcing feedback loop.

By developing these points, the paper extended existing critical research on marketing algorithms (Zwick and Denegri Knott, 2009; Beckett, 2012; Cluley and Brown, 2015) and studies on how people assume a debt-tolerant, self-disciplined financial subjectivity that encourages them to borrow and pay their debt on time (Langley, 2008, 2009) by complementing the current focus on governmentality and subjectivity formation with an analytical angle on processes of class formation. The paper suggested, first, that the processes of subjectivity formation identified by these literatures are implicated in the shaping of the cultural element of class. As marketing targets different discourses and devices at different segments, it calls forth different kinds of subjectivities and self-disciplining practices for consumers that are categorized into different segments by algorithms. Second, the paper extended the focus of these works on subjectivity to the economic element of class, by showing how the marketing of financial products may contribute to patterns of *economic* inequalities.

Finally, the paper added to the performativity literature on marketing’s society-shaping role. Prior literature has theorized how marketing shapes social groups, particularly the cultural element of class and lifestyles (Ariztía, 2014; Jacobi et al., 2015). Albeit this literature did not look at algorithmic sorting and matching, recent debates in critical marketing studies touched on the question of whether algorithmic segmentation and targeting used in marketing still performs social groups; or rather, in the era of ‘dividual’ consumers and the perpetual re-matching of consumers with products in large-scale databases, marketing no longer assumes nor performs social groups.

The paper did not take a position in this debate. How a specific sorting and matching mechanism of marketing affects inequalities is an empirical question. The extent to which the segments overlap or crosscut traditional markers of inequalities, and the characteristics of the products that are matched with them determines how marketing sorting practices deepen or flatten existing class inequalities or create potentially new ones. Albeit this paper discussed several examples, its aim was not to provide an answer to this empirical question. Instead, the two-fold framework aimed to provide an analytical lens through which these processes can be usefully thought through and to promote more focused research into how marketing algorithms contribute to social inequalities. A key point of this framework is that segmentation and eligibility categories are not neutral, but political (in the traditional sense of having power) and largely consequential for producing objective life chances – and thus, inequalities. Being in charge of segmentation, product development, targeting and user experience design, marketing thus may acquire a stronger society-shaping power than before.

In analysing these processes, the paper cautioned against interpretations that – faced with the complexity and transience of algorithm-generated segments – suggest too easily that traditional categories of class are no longer relevant criteria of classification. They may not be evident at first glance in the current segmentation trends, based on behaviours, needs and assumedly unbiased machine learning-generated segments. As the examples have shown, many of these practices are class-blind. They do not use the language of class, but they use the objective markers of traditional class categories: class variables of income, living location and education, as well as proxies of class, such as payment behaviour. However, by matching low-income segments with products with worse conditions and scripts that encourage short-term planning and day-to-day survival, while high-income segments with ‘reward’ products and scripts of long-term planning and wealth accumulation, even class-blind algorithms may reproduce and deepen existing class inequalities.

The paper opens up several areas of further research: (1) Consumers as *objects* of algorithms: How are consumers categorized by algorithms for different marketing problems (e.g. product development, ad targeting)? To what extent do algorithms use traditional class variables or their proxies? If they use other variables, what kind of new ‘classification situations’ and new inequalities do classifications based on these variables produce? What kind of products do they offer for the different categories and what is the potential effect of this matching on inequalities (deepening, flattening, cross-cutting)? What kind of discourses are offered for different categories and to what extent do they call forth different subjects and governance mechanisms? (2) Consumers as *subjects* of algorithms: What kind of decisions are made on behalf of consumers by algorithms? What are the assumptions of consumer subjectivity that underlie these decisions? Do they differ depending on how the consumer is categorized? To what extent do these algorithmic choices contribute to cultural and economic differences across social groups? (3) *Limits* to the class-shaping power of marketing: To what extent are marketing categorizations independent or limited by other classifications used by companies? For example, to what extent do marketing categories need to follow credit scoring categories? (4) *Differences* across markets: How do these processes differ across markets? For example, in what product categories does marketing shape economic inequalities, cultural inequalities or both? Answering these questions would allow us to map how exactly marketing shapes inequalities and to understand its power to shape social classes.

## Notes

1. We define algorithms as calculation-based decision rules as opposed to other forms of decision-making based on, for example, intuition or heuristics. In this sense, even a simple Excel command that calculates marks based on interim exam scores is an algorithm.

2. Google is working on a new tracking system that would eliminate third-party cookies and would group people with similar browsing histories into groups for interest-based advertising (BBC, 2021). What kind of data would be included in creating the groups and how this data would be combined with other online data is not known at the time of writing.
